# Laser-Guided Sperm Selection: Optimizing the Reproductive Success Rate in Assisted Reproductive Technology

**DOI:** 10.7759/cureus.49052

**Published:** 2023-11-19

**Authors:** Charu Pareek, Ujwal Gajbe, Pranita A Bawaskar, Gulshan R Bandre, Ankit K Badge

**Affiliations:** 1 Clinical Embryology, Datta Meghe Medical College, Datta Meghe Institute of Higher Education and Research (Deemed to Be University), Nagpur, IND; 2 Anatomy, Datta Meghe Medical College, Datta Meghe Institute of Higher Education and Research (Deemed to Be University), Nagpur, IND; 3 Microbiology, Jawaharlal Nehru Medical College, Datta Meghe Institute of Higher Education and Research (Deemed to Be University), Wardha, IND; 4 Microbiology, Datta Meghe Medical College, Datta Meghe Institute of Higher Education and Research (Deemed to Be University), Nagpur, IND

**Keywords:** assisted reproductive technology, zeta potential, magnetic activated cell sorting, intracytoplasmic morphologically selected sperm injection, azoospermia, laser-assisted sperm selection

## Abstract

Assisted reproductive technologies (ART) enable these patient’s spermatozoa to fertilize the oocyte and create viable and healthy offspring, but the effectiveness of the various procedures still has room to increase. In the field of assisted reproductive technology, the need to improve fertility results has led to the development of novel sperm selection strategies. Laser-assisted selection of immotile sperm (LAISS) appears to be a promising strategy, harnessing the power of modern optical instruments to better the selection process and, ultimately, maximize the probability of successful fertilization. This technology takes advantage of sperm cells' distinctive features, such as shape, form, and motility patterns, that can be sensitively changed by laser forces. Using precision laser manipulation, spermatozoa with desirable features can be precisely targeted, improving the overall quality and viability of the sperm population. The existence of an elevated percentage of DNA-damaged sperm in a patient's ejaculation may be one of the key factors decreasing ART outcomes. As a result, one of the most difficult tasks in reproductive medicine is ensuring the best quality of spermatozoa utilized in ART, particularly with regard to genetic integrity. The most recent approaches for preparing and selecting human spermatozoa by LAISS techniques are covered here, with an emphasis on those that have been shown to improve.

## Introduction and background

Approximately 10 to 20% of male individuals dealing with infertility encounter azoospermia. When sperm retrieval requires surgery, motility is often limited [1]. In the process of intracytoplasmic sperm injection (ICSI), embryologists may encounter a challenge when dealing with fully immotile sperm samples. Fertilization can still occur with immotile sperm, indicating that sperm viability is more important for successful fertilization. Consequently, the issue of selecting a live sperm from a pool of immotile sperm on the day of oocyte retrieval for ICSI is of paramount importance [2]. Injection of immotile spermatozoa from ejaculates or biopsies has resulted in pregnancies and healthy offspring [3]. Reports suggest that fertilization and embryo utilization rates are lower in ICSI with immotile spermatozoa than in motile ones. To offer patients convenient and cost-effective treatments, laboratory techniques must be used to differentiate between viable but immotile and deceased spermatozoa [4,5].

For assessing the viability of immotile sperm, the hypo-osmotic swelling test, chemical induction of tail movement, and laser techniques have been developed. Laser-assisted selection for immotile spermatozoa in ICSI leads to better outcomes than conventional selection methods. ICSI has achieved comparable fertilization rates and embryo cleavage using immotile spermatozoa selected through laser assessment compared to the utilization of motile testicular spermatozoa [6]. This review article aims to evaluate the role of laser-guided sperm selection in assisted reproductive technology and assess its impact on optimizing the success rate of reproductive procedures.

## Review

Sperm morphology was hypothesized to be a significant factor in predicting fertility, and it was found to be closely connected to fertilization and conception rates in the naturally occurring fertilization process as well as in the procedure of intrauterine insemination and traditional in vitro fertilization (IVF) procedures [7]. For sperm to successfully cross the zona pellucida and fuse with the oocyte's plasma membrane, they must have normal morphology [8].

Laser-assisted selection of immotile sperm 

**Figure 1:**
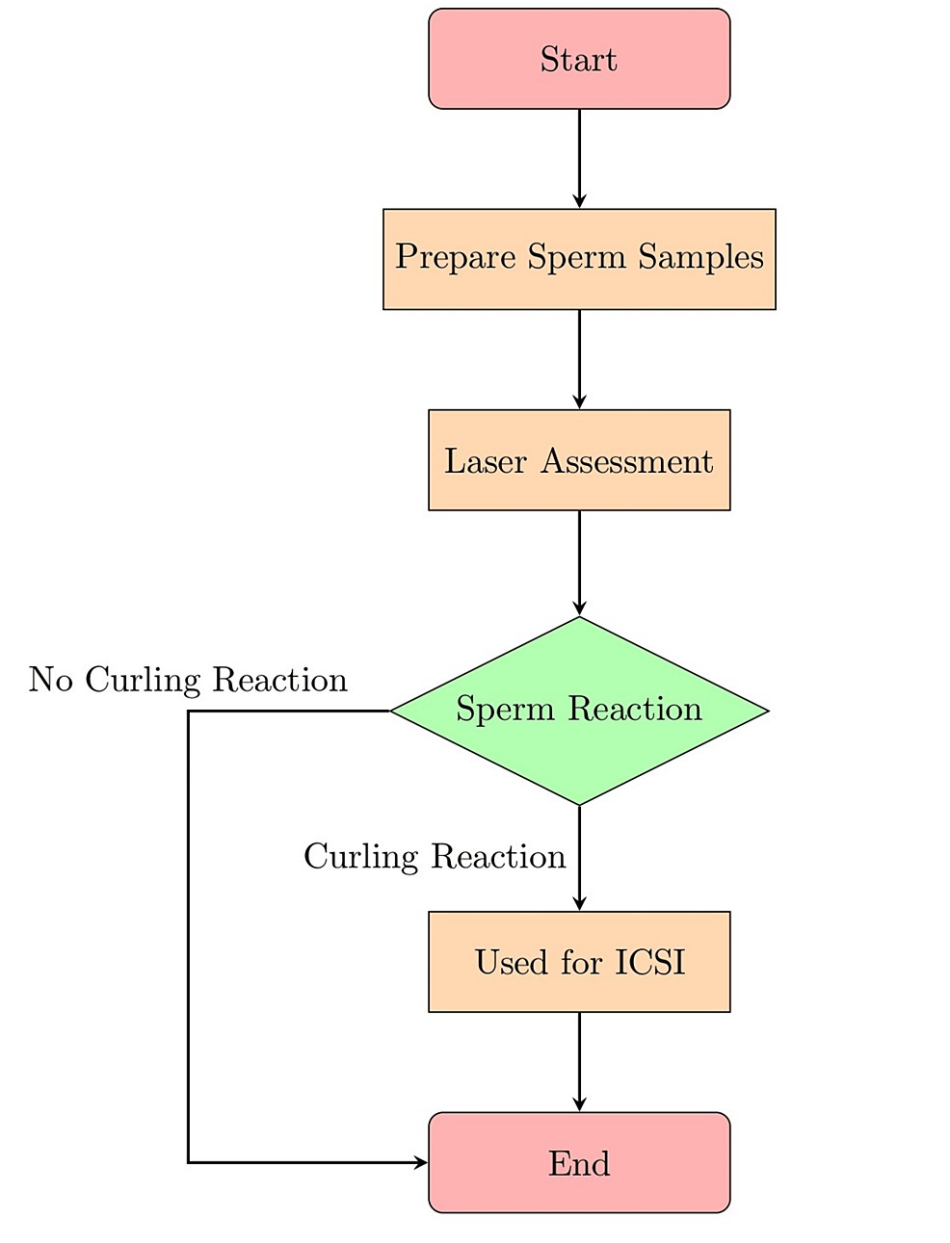
Flowchart illustrating the process of laser-assisted selection of immotile sperm ICSI- Intracytoplasmic sperm injection Credit: The flowchart was prepared by the author from the process adapted from references [11,12].

Laser-assisted selection of immotile sperm (LAISS) makes it possible to precisely identify and choose healthy sperm with ideal morphology and undamaged DNA. Successful fertilization and the development of embryos are greatly increased by concentrating on healthy sperm. It is a non-invasive technology, in contrast to conventional methods that call for invasive treatments. This lessens patient discomfort while lowering the chance of complications from more intrusive procedures [9]. Previously, immotile sperm have been demonstrated to become more motile after laser activation. For couples who are struggling with infertility due to immotile sperm, this is a great advancement. Producing healthy embryos and lowering the risk of genetic problems in progeny can help select sperm with fewer congenital abnormalities. Ethical questions come up with any modern reproductive technique [10]. Concerns about the long-term impacts on offspring and the possibility of unforeseen repercussions call for further study and careful consideration. Even though LAISS has produced encouraging results, individual instances can affect success rates, and other research is required to hone the method and ensure constant efficacy. A single laser shot to the sperm tail generates a curling reaction in apparently viable spermatozoa while causing no reaction in presumably dead spermatozoa, making the selection of apparently viable spermatozoa simple and effective. The procedure is simple to carry out in a standard culture medium, and the spermatozoa can be employed immediately for ICSI [11,12]. The process of LAISS is shown in Figure 1.

Intracytoplasmic morphologically selected sperm injection

The remaining sperm fraction chosen for microinjection in individuals choosing ICSI treatment was the first area to get intracytoplasmic morphologically selected sperm injection (IMSI) treatment. It was only used on a fraction of motile spermatozoa. Additionally, since sperm cells with severe abnormalities are not used for microinjection, which are already visible at low magnification were disregarded [13]. The morphological state of six subcellular organelles, including the acrosome, post-acrosomal lamina, neck, mitochondria, tail, and nucleus, was assessed for each sperm cell. Certain subcellular organelles were deemed to be morphologically aberrant based on specific abnormalities. The chromatin mass shape and appearance are used to evaluate the morphology of the sperm nucleus. The typical sperm nucleus is oval, smooth, and symmetrical and exhibits a uniform chromatin mass devoid of extrusions or invaginations [14]. Additionally, the nucleus only has a single vacuole that occupies 4% of the nuclear surface. The base of a normal head is U-shaped. If the acrosome and post-acrosomal lamina are partially present, vesiculated, or have disappeared, then sperm is considered abnormal. An abaxial deviation, cytoplasmic droplets, or other diseases cause neck anomalies. The tails of a typical spermatozoon shouldn't be broken, short, double, or coiling. Abnormalities of mitochondrial sperm can be seen when sperms are missing or partially developed [15].

Magnetically activated cell sorting

A great method for separating cells of interest from mixed cell populations is magnetic-activated cell sorting (MACS). The sorter uses magnetic micro- and nanoparticles coupled with antibodies particular to the desired cell membrane protein [16]. The cells attached to magnetic particles are in a region of strong magnetic energy gradients and gradually alter their course [17]. Unbound cells maintain their initial routes and are not impacted by the magnetic field. Cells can be enhanced with MACS, either with or without phosphatidylserine (PS) exposed to their surfaces. The presence of PS on the cell surface increases the binding efficiency of MACS beads, allowing for more precise cell separation. The enhanced cells can then be directed toward specific regions in the body using magnetic fields, enabling targeted drug delivery or tissue regeneration. Annexin V microbeads are used to magnetically label cells as they are processed along a MACS column in a MACS separator's magnetic field. This allows for isolating specific cell populations based on their surface markers. The magnetic labeling with Annexin V microbeads provides a highly efficient and specific method for cell separation. The MACS separator's magnetic field allows for easy and gentle separation of the magnetically labeled cells while the unbound cells continue along their original route unaffected. Unlabeled cells move through the column while the magnetically tagged and PS-exposing cells remain the same. When the magnetic field has been removed from the column, the magnetically trapped PS-exposing cells may be evacuated as the positively chosen cell fraction. Prior studies have shown that mature sperm fractions exhibit higher nuclear maturity and a decreased incidence of apoptosis [18].

Zeta potential

The Zeta technique of sperm processing allows quick recovery of sperm with improved sperm characteristics, including normal morphology, DNA normal integrity, and aniline blue maturity. These factors are linked to enhanced sperm intracytoplasmic injection, fertilization, and pregnancy [19]. Additionally, sperm progressive motility and hyperactivation were increased, indicating either a brief exposure to the serum-free state or the modification from the attaching-detaching process served as a trigger to accelerate sperm metabolic activity without prematurely inducing acrosome reactions [20]. After assisted reproductive technologies (ART) operations, increased progressive motility and hyperactivation were indicators of successful pregnancies. Total sperm motility remained constant, in contrast to the aforementioned metrics, indicating that there is no correlation between total motility and zeta potential [21].

Discussion

Aktan et al. [22] concluded that laser sperm contact can identify viable but immotile spermatozoa. The utilization of viable sperm that can promote oocyte activation and embryonic development is required for the effective execution of ICSI. Spermatozoa are chosen based on motility, a clear indicator of viability [22]. However, in cases of total asthenozoospermia, morphological selection of viable sperm is impossible. Just a single laser shot to the distant end of the sperm tail elicits curling of the sperm tail in only the viable sperm, which is comparable to the reaction seen in the hypo-osmotic swelling test. High fertilization and cleavage rates using this procedure for sperm selection before ICSI for cases having immotile sperm in initial testicular biopsy material as well as cases with ejaculated immotile sperm [23].

Antinori et al. [24] discussed the benefits of a new enhanced ICSI technique, IMSI, in managing patients with severe oligoasthenoteratozoospermia (OAT). IMSI was used in the new approach, which was based on an initial motile sperm organellar morphological evaluation at x6600 high magnification. Irrespective of the number of previous IVF failures, IMSI is much more advantageous than ICSI for all individuals with severe OAT [24]. Considering a poor reproductive prognosis, cases with two or more failed efforts appear to profit from the statistically significant double in their pregnancy rate as well as a surprising reduction of the miscarriage rate. IMSI could be suggested as a routine IVF approach to solving complex male infertility patients from the first try, with proper standardization of the procedure in order to acquire knowledge of the selection criteria as well as eliminate certain of the above practical obstacles. The MACS approach selects spermatozoa with damaged membranes and PS externalization as a symptom of death at the molecular level, supplementing the sperm preparation routine in ART [25]. There have been very few studies that assess the efficacy of sperm separation with apoptosis utilizing the MACS approach in ICSI cycles for patients using their own and donated eggs. When treated sperm is administered to good-quality eggs, the efficiency of annexins in removing apoptotic sperm is demonstrated. These findings have positive implications and suggest that employing this approach could enhance the total number of clinical conceptions [26]. Kheirollahi-Kouhestani et al. [27] discussed that zeta sperm selection for assisted reproduction ought to attempt to reduce the aberrant sperm participating in the fertilization process. Non-viable spermatozoa, leukocytes, bacteria, and other sources of contamination should be eliminated in the optimal sperm-separation procedure. It should be rapid, simple, and inexpensive, with the ability to eliminate as many mature spermatozoa as feasible without causing sperm damage. Zeta sperm selection looks to be an easy and affordable process for selecting viable sperm that have minimal DNA damage and do not require any additional instruments. Sperm picked using the Zeta approach might have less DNA fragmentation than sperm selected while using standard density gradient centrifugation. As a result, combining these two treatments could enhance the viability of the sperm chosen for ICSI and result in better pregnancy rates [27]. Sperm selection methods, management, and outcomes are as follows (Table 1).

**Table 1:** Sperm selection methods, management, and its outcomes ICSI: Intracytoplasmic sperm injection; IMSI: Intracytoplasmic morphologically selected sperm injection; HOS: hypo-osmotic swelling; MACS: Magnetic activated cell sorting; DNA: deoxyribonucleic acid; OAT: oligoasthenoteratozoospermia

Author	Prognosis	Management	Outcome
Aktan et al. [22]	Use of laser in case of total immotile	Prior to ICSI, a laser pulse was administered along with the HOS test.	The take-home baby rate per cycle was 19% compared to 5.9% for sperm extracted from the testicles, and 28% compared to 16.7% for ejaculated sperm that underwent laser selection.
Antinori et al. [24]	Use of severe OAT	Individuals experiencing severe infertility who underwent IMSI displayed markedly increased clinical pregnancy rates compared to those who underwent standard ICSI.	Patients with severe OAT experience enhanced pregnancy rates through IMSI compared to traditional ICSI.
Merino-Ruiz et al. [26]	Use of MACS to select non-apoptotic sperm	MACS resulted in higher fertilization rates among couples using self-derived oocytes and greater clinical pregnancy rates among those using donated oocytes.	In cases involving donated oocytes, MACS enhances both fertilization and clinical pregnancy rates.
Kheirollahi-Kouhestani et al. [27]	Use of zeta potential to reduce DNA fragmentation in infertile male	Sperm DNA fragmentation Chromatin protamination	Utilizing Zeta potential for sperm selection resulted in notable reductions in the percentage of DNA-damaged sperm and chromatin condensation.

Enhancing laser-assisted selection of immotile sperm technique

One area for advancement is the creation of more compact and portable LAISS devices. This would make it easier for fertility clinics to undertake the operation and broaden its availability to locations with limited resources [28]. Portable LAISS devices may be especially useful in remote or underserved areas, bringing modern reproductive therapies to a larger population. By incorporating cutting-edge imaging and analyzing technology precisely into the LAISS apparatus, real-time information regarding sperm condition and characteristics may be provided. High-resolution microscopes and computer vision algorithms could be used to examine sperm morphology, DNA integrity, and other crucial variables [29]. This type of integration would allow fertility professionals to make more educated decisions about sperm selection. Robotics could improve precision and consistency by automating certain parts of the LAISS method. Robotic devices could manage the laser target and sperm manipulating processes, reducing the possibility of human error and outcome unpredictability. This ensures that the laser is precisely directed to the appropriate sperm cells, hence enhancing the selection process [30].

Creating real-time feedback mechanisms to assess the impact of laser stimulation on sperm motility may allow for dynamic modifications during the operation. Specialists could fine-tune parameters to get the best potential motility improvement without causing injury by obtaining immediate information on the reaction of sperm to laser stimulation [31]. Integrating analytical abilities into LAISS devices may allow for a more in-depth review of treatment results over time. Clinicians could discover patterns, enhance treatment protocols, and tailor procedures for particular patients based on history by collecting and evaluating data from repeated treatments [32].

## Conclusions

LAISS has altered the way to address sperm selection by using the efficacy of laser technology and sophisticated microscopy, opening the possibility of improved fertilization outcomes and increased odds of successful conception. LAISS approach marks a significant advancement in the area of assisted reproductive technology, opening up a new vista of hope for couples struggling with male factor infertility. This technique has proven extremely effective at improving sperm quality and achieving successful fertilization. There is ample evidence supporting the enhancement of sperm motility and DNA integrity using laser-assisted sperm selection. This advanced technique allows scientists to selectively target and manipulate individual sperm cells based on their physical characteristics, such as size, shape, and motility. By precisely isolating the healthiest and most viable sperm, LAISS significantly improves the chances of successful fertilization and, ultimately, the birth of a healthy baby. With further research and advancements in this field, LAISS has the potential to revolutionize reproductive medicine.
